# Fast and Fractionated: Correlation of Dose Attenuation and the Response of Human Cancer Cells in a New Anthropomorphic Brain Phantom

**DOI:** 10.3390/biomimetics10070440

**Published:** 2025-07-03

**Authors:** Bernd Frerker, Elette Engels, Jason Paino, Vincent de Rover, John Paul Bustillo, Marie Wegner, Matthew Cameron, Stefan Fiedler, Daniel Häusermann, Guido Hildebrandt, Michael Lerch, Elisabeth Schültke

**Affiliations:** 1 Department of Radiooncology, Rostock University Medical Center, 18059 Rostock, Germany; bernd.frerker@med.uni-rostock.de (B.F.); guido.hildebrandt@med.uni-rostock.de (G.H.); 2 Australian Synchrotron/ANSTO, Clayton, Melbourne, VIC 3168, Australia; elette@uow.edu.au (E.E.); cameronm@ansto.gov.au (M.C.); danielh@ansto.gov.au (D.H.); 3 Centre of Medical Radiation Physics, University of Wollongong, Wollongong, NSW 2522, Australia; jrp933@uowmail.edu.au (J.P.); vdr@uowmail.edu.au (V.d.R.); jpob066@uowmail.edu.au (J.P.B.); mlerch@uow.edu.au (M.L.); 4 Institute of Product Development and Mechanical Engineering Design, Hamburg University of Technology, 21073 Hamburg, Germany; marie.wegner@tuhh.de; 5 European Molecular Biology Laboratory (EMBL), Hamburg Outstation, 22607 Hamburg, Germany; sfiedler@embl-hamburg.de

**Keywords:** anthropomorphic phantom, biomimetic tool, brain, human cancer cell lines, microbeam radiation therapy (MRT), spatial dose fractionation (SFRT), synchrotron

## Abstract

The results of radiotherapy in patients with primary malignant brain tumors are extremely dissatisfactory: the overall survival after a diagnosis of glioblastoma is typically less than three years. The development of spatially fractionated radiotherapy techniques could help to improve this bleak prognosis. In order to develop technical equipment and organ-specific therapy plans, dosimetry studies as well as radiobiology studies are conducted. Although perfect spheres are considered optimal phantoms by physicists, this does not reflect the wide variety of head sizes and shapes in our patient community. Depth from surface and X-ray dose absorption by tissue between dose entry point and target, two key parameters in medical physics planning, are largely determined by the shape and thickness of the skull bone. We have, therefore, designed and produced a biomimetic tool to correlate measured technical dose and biological response in human cancer cells: a brain phantom, produced from tissue-equivalent materials. In a first pilot study, utilizing our phantom to correlate technical dose measurements and metabolic response to radiation in human cancer cell lines, we demonstrate why an anthropomorphic phantom is preferable over a simple spheroid phantom.

## 1. Introduction

Spatially fractionated irradiation techniques have gained increasing clinical attention over the last years, and the first human patients have been successfully treated [1,2]. A significant reduction in tumor volume, accompanied by an equally significant improvement in the patients’ quality of life, has been achieved in a pilot study of microbeam radiation therapy (MRT), treating canine patients with primary malignant brain tumors [3,4]. Considering that these results have been obtained after only one single irradiation session, with no prior removal of the tumor bulk, it seems reasonable to consider the first clinical MRT studies in human patients.

In the process of creating quality assessment tools for this new irradiation technique, we have considered an anthropomorphic shape and the composition of tissue-equivalent materials as especially important. While perfect spheres are considered optimal phantoms by physicists, this does not reflect the wide variety of head sizes and shapes in our patient community. The particular shape of the skull determines the depth of an irradiation target from the surface. Depth from surface is a key parameter in medical physics planning. Another important parameter in treatment planning is the dose attenuation by the tissue between dose entry and irradiation target. Thus, the design of an optimal phantom should incorporate biomimetic principles, considering the thickness of the bone (which varies widely within each individual skull), and be composed of materials with dose absorption qualities similar to those of organic tissues. Phantoms designed according to biomimetic principles have recently been used by other researchers to optimize the reconstruction of skull defects [5] and to facilitate neurosurgical training [6].

To investigate X-ray dose deposition in still experimental radiotherapy techniques, we initially used a commercially available anthropomorphic slice phantom [7]. This Alderson phantom, produced from tissue-equivalent materials, is frequently used for dose measurements in medical imaging [8]. It contains a series of axial slices, each approx. 25 mm high, with pre-defined vertical hollows which can accommodate probes for dose measurement, such as a microDiamond (PTW, Freiburg, Germany). The unused hollows can be filled with pegs provided with the phantom. We used some of these channels to insert biological samples. One of the disadvantages in working with the Alderson phantom was our dependency on pre-defined positions of the hollows, which did not always suit the geometry of our experiment. A second disadvantage was that only one phantom slice for each irradiation location was available. The availability of more than one slice of the same type would allow pre-loading with samples and thus a much faster sample throughput, resulting in a decrease in labor hours. Last but not least, the microDiamond, which is an essential dosimetry tool in synchrotron radiotherapy, did not fit easily into the hollows of the Alderson phantom. We therefore decided to produce our own brain phantom, matched to an Alderson brain slice at the level of the pituitary gland.

By slightly modifying our approach, it can even contribute to an individualization of the treatment concept: phantoms can be generated based on a patient’s CT scan to match patient-specific key parameters of X-ray beam attenuation, such as the shape of the head and tissue structures in the region of interest. A CT scan of this phantom can be uploaded into the treatment planning system and integrated with different treatment plan versions to illustrate the dose distribution across the biological samples used for radiobiology studies, aiming to identify the optimal treatment plan. This approach can reduce radiobiological uncertainty in future clinical studies involving new radiotherapy techniques.

## 2. Materials and Methods

### 2.1. Producing the Biomimetic Tool

The aim was to produce a phantom which represented shape and tissue distribution similar to that normally found in a human head. In addition, the materials should absorb X-rays in a similar way as seen in organic tissue. Different from commercially available brain slice phantoms [8,9], it should be optimized to fit the requirements of our radiobiology study, including the size of dosimetry probes and sample containers.

**Figure 1:**
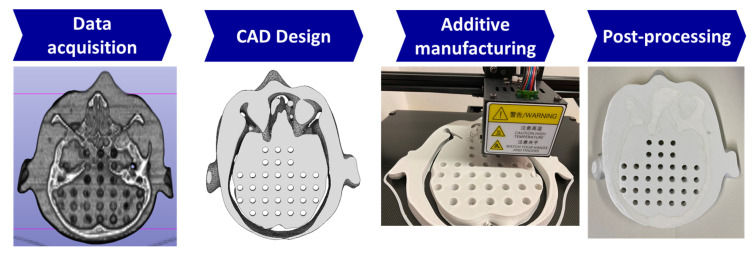
Workflow for the custom additive manufactured brain slice based on the Alderson phantom at the level of the pituitary gland.

To produce the brain slice phantom, a CT scan of the Alderson phantom slice at pituitary gland level was obtained at a Brilliance CT Big Bore Oncology scanner (Philips, Hamburg, Germany) operating at 290 mA and 120 kV. The scan was segmented using the open-source software 3D Slicer [10]. The segmented model was smoothed and further processed in CAD software (Autodesk Inventor by Autodesk, Inc., San Rafael, CA, USA). The bone structures were intentionally left as voids during design to allow for post-manufacturing filling to simulate bone structure. The phantom was printed using a fused deposition modeling (FDM) 3D printer, the Ultimaker S5 (Ultimaker B.V., Geldermalsen, The Netherlands) and polylactic acid (PLA) filament (Das Filament, Braunschweig, Germany). The 3D-printing parameters were set to 0.15-layer height, 2.85 mm filament thickness, and 0.4 extrusion width. The printer achieves printing accuracy of ±0.3 mm. The density of PLA is very similar to that of brain tissue, as demonstrated previously [11]. Furthermore, the material can be modified to represent a range of different soft tissue densities [12]. During post-processing, the support structures were removed, and the hollow cavities designated for bone were filled with a bone surrogate mixture composed of silicone and gypsum (Figure 1) with a density similar to that of bone. Pegs were manufactured to fill the unused 7 mm holes.

**Figure 2:**
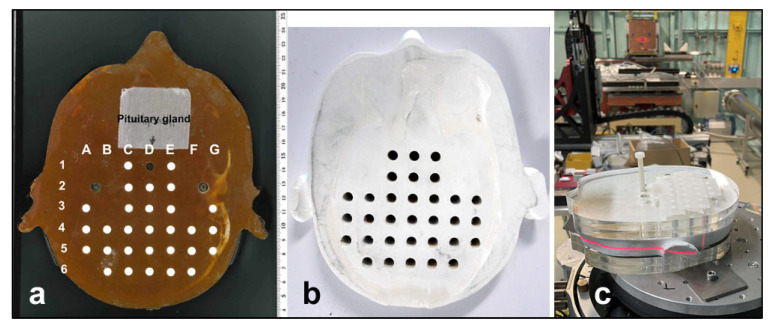
Brain slice of an Alderson phantom at the level of the pituitary gland, overlaid with a virtual grid structure for reproducible identification of biological samples (**a**). In-house produced brain phantom slice (**b**) and setup of the in-house brain phantom at the IMBL beamline (**c**). The horizontal red laser runs over the slice produced in modification of the Alderson phantom.

To allow reproducible identification of each biological sample, we overlaid the brain slice with a virtual grid structure (Figure 2a). The two slices above and below the targeted brain slice (Figure 2b) were replaced by 25 mm thick slices of plexi, in order to simulate the scatter conditions within a larger brain volume (Figure 2c).

### 2.2. Cancer Cell Cultures

The aim of this part of the study was to understand the biological response to irradiation as a function of depth from surface, similar to that seen in the head of human patients. While this type of phantom is lacking the complex biological feedback mechanisms seen in living organisms, its meaningfulness with regard to the real-life experience should be far superior to a simple irradiation study where samples are irradiated at one single pre-defined position, without interference by other biological materials of varying density. Tissue density determines the extent of X-ray absorption between dose entry and irradiation target and is therefore a key parameter in medical physics therapy planning.

For our study in anthropomorphic phantoms, we have used two different commercially available human cancer cell lines: one malignant primary brain tumor cell line (U87, also known as HTB-14; ATCC, Manassas, VA, USA) and one lung cancer cell line (A549, also known as CCL-185; ATCC, USA). The A549 cell line was chosen for this evaluation because lung cancer is the most common cause of cancer death and it is a common cause of metastatic disease in the brain [13]. Cells were proliferated, passaged at least once and harvested.

We have tested two approaches to presenting cancer cells for irradiation: 

Cells distributed throughout a 3D agar gel column, similar to 3D distribution within a tumor: For this method, agar (catalogue number 4508.1, Carl Roth GmbH, Karlsruhe, Germany) was heated to below boiling point in sterile filtrated water, prepared at a concentration of 1%. The clear agar suspension was allowed to cool down and mixed with the cancer cells suspended in clear DMEM growth medium, supplemented by 10% fetal bovine serum and 1% (*v*/*v*) Penicillin–streptomycin–glutamine (catalogue number 10378016, Gibco, Life Technologies, Grand Island, NY, USA), to achieve a cell concentration of approx. 1 million cells per ml in a final agar concentration of 4%. This suspension was aliquoted into small tubes, which were 3D-printed from polylactic acid+ (PLA+) polymer filament (eSun, Shenzhen, China), to fit into the hollows of our brain slice phantom. The agar was allowed to solidify in the tubes at room temperature for 1 h. Prior to the cell culture study, the material had been tested for potential cell toxicity but was found not to impair cell proliferation. This approach was used for work in U87 brain cancer cells.

Cells compacted into a cell pellet: The cell suspension was aliquoted into 0.2 mL PCR tubes and centrifuged at 1000 rpm for 5 min at room temperature, with the aim to form a pellet for irradiation. The supernatant was not removed for the irradiation procedure. This approach was used in experiments with A549 lung cancer cells, where clonogenic colony formation was one of the analytic methods chosen.

### 2.3. Simulation for Depth-Dependent Dose Deposition After MRT

Reference dosimetry was performed in a 100 mm × 100 mm × 100 mm Perspex^TM^ solid water phantom using a PTW PinPoint 3D Ionization Chamber (31022, Freiburg, Germany). With a sensitive volume of 0.016$\;cm^{3}$, the PinPoint IC is certified for broad-beam field sizes as small as $20\times 20\;mm^{2}$, making it the ideal candidate for small-field and small-animal irradiation [14]. The entrance dosimetry protocol used for this work was developed at DESY (Hamburg, Germany) and is adapted from that used at the ESRF (Grenoble, France) [15]. The entrance dose was defined at an equivalent measurement depth of 5 mm. Here, the measurement was performed in the DESY entrance reference phantom, which is a 50 mm × 50 mm × 50 mm Perspex^TM^ phantom using the PTW PinPoint 3D IC, with the central axis of the IC at 5 mm depth.

For microbeam characterization, the PTW microDiamond detector (60019, Freiburg, Germany) was cross-calibrated to the PTW PinPoint chamber under the same entrance reference phantom measurement conditions. With a minimal cross-sectional area of $1.1\times 0.001\;mm^{2}$, the PTW microDiamond is ideal for microbeam characterization [16,17].

Geant4 (version 11.02) dose simulations were performed using the previously validated DoseMRT software package (version 1.1) [18]. The software package was used to import a CT scan of the 3D-printed brain slice phantom and convert it into a voxelized phantom with custom defined materials based on the known composition of the phantom. Experimental peak dose measurements in the surface reference phantom were used to calibrate the peak dose in the simulation results.

### 2.4. Dosimetry and Microbeam Irradiation at the Synchrotron

Microbeam irradiation studies were conducted in Hutch 2B of the Imaging and Biomedical Beamline (IMBL) of the Australian Synchrotron (AS) at a ring current of 200 mA. Before irradiation, reference dosimetry was performed using a PinPoint 3D Ionization Chamber with a sensitive volume of 0.016$\;cm^{3}$ (PTW, Freiburg, Germany), according to a protocol used at the European Synchrotron Radiation Facility (ESRF, Grenoble, France), adapted for use at IMBL [15]. For microbeam characterization, a microDiamond detector (PTW, Freiburg, Germany) was cross-calibrated to the PTW PinPoint chamber under the same conditions. The dosimetry procedures have been described in more detail previously [19].

To generate the microbeam arrays for irradiation, a multislit collimator (MSC) was inserted in the incident beam at IMBL, resulting in quasi-parallel microbeams at the treatment position, where the individual microbeam width was 50 µm, spaced at a center-to-center distance of 400 µm. The MSC has been characterized previously [20].

Broad-beam irradiation at FLASH dose rates (>40 Gy/s) was conducted with a skin- entrance dose of 50 Gy, at a dose rate of 226.53 Gy/s. Spatial dose fractionation was conducted as unidirectional microbeam irradiation, with MRT peak doses of 50 Gy and 400 Gy, respectively, at skin entrance. The corresponding MRT valley doses were 1.07 Gy and 8.59 Gy, respectively. Dose deposition occurred at a dose rate of 172.34 Gy/s (comparison clinical radiotherapy: 6–10 Gy/min).

### 2.5. Colony Counts and WST Test

Resuspending cells irradiated while distributed in agar: After irradiation, each tube containing cancer cells distributed three-dimensionally within an agar column was dropped into a 1.5 mL Eppendorf tube containing clear DMEM/F-12 (DMEM without phenol red as pH indicator, gibco, catalogue number 11039-021) and placed in a standard incubator for 2 h. This facilitated the next step, pushing the agar column containing the cells out of the tube and into the DMEM. The agar column was homogenized, and the suspension thus generated was centrifuged at 1000 rpm for 5 min at room temperature. The supernatant, containing the dissolved agar, was aspirated, and the cells were resuspended in DMEM and counted.

Resuspending cells irradiated as pellet: Each cell pellet was, after irradiation, resuspended in 200 µL of clear growth medium and then transferred into a 1.5 mL Eppendorf tube already containing 800 µL of clear DMEM/F-12.

Colony counts: T25 flasks were seeded for colony counts with 400 cells/flask. Colonies were terminated 10 days after irradiation. The colonies were fixed with 70% alcohol and stained with crystal violet.

WST: The WST test provides a simple and accurate method to assess metabolic activity as the equivalent of cell proliferation. In a one-step procedure, the reagent detects the cleavage of the tetrazolium salt WST-1 (4-[3-(4-Iodophenyl)-2-(4-nitro-phenyl)-2H-5-tetrazolio]-1,3-benzene sulfonate) to formazan by mitochondrial dehydrogenase. The absorbance of the formazan dye is detected at a wavelength range of 420–480 nm, where an increase in viable cells is reflected in an increase in formazan dye production. At the available plate reader (Muse Biotek Synergy, Agilent, Santa Clara, CA, USA), we read the absorbance at 450 nm, with a reference wavelength of 620 nm.

**Figure 3:**
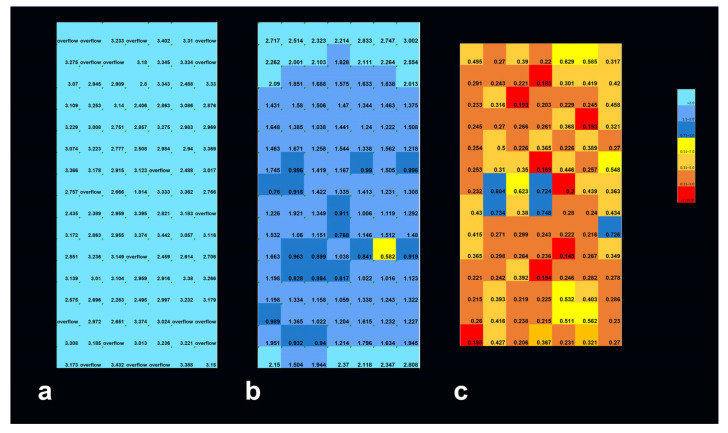
WST signal (arbitrary units) representing metabolic intensity in human lung cancer cells (A549), seeded in 384-well plates: non-irradiated controls (**a**) and samples irradiated in unidirectional MRT mode with peak doses of 400 Gy, at 48 h (**b**) and 5 days after irradiation (**c**). In the samples analyzed 48 h after MRT, a slight decrease in metabolic activity is already seen in the center of the irradiation field. At 6 days after MRT, the decrease in metabolic activity is already very obvious across the entire field of irradiation. Smaller numbers equal lower metabolic activity.

For WST in cells recovered from an agar column after irradiation (U87 human brain cancer cells), approx. 5000 cells/well were seeded. For WST recovered from cells irradiated as pellet (A549 human lung cancer cells), approx. 2000 cells/well were seeded into 96-well plates in triplicates. The volume in each well was topped up to 100 µL per well with clear growth medium to obtain a good WST signal, because WST is a colorimetric test in the red spectrum (catalogue number 05015944001, Roche Diagnostics GmbH, Mannheim, Germany). Following the results of a preparatory experiment, where human lung cancer cells (A549) were irradiated in 384-well plates, we would expect to see increasingly distinct value separation between irradiated and non-irradiated cells with longer intervals between irradiation and readout (Figure 3). The samples were read 20 min (lung cancer) and 1 h (U87 glioma) after adding 10 µL of reagent to each sample.

## 3. Results

**Figure 4:**
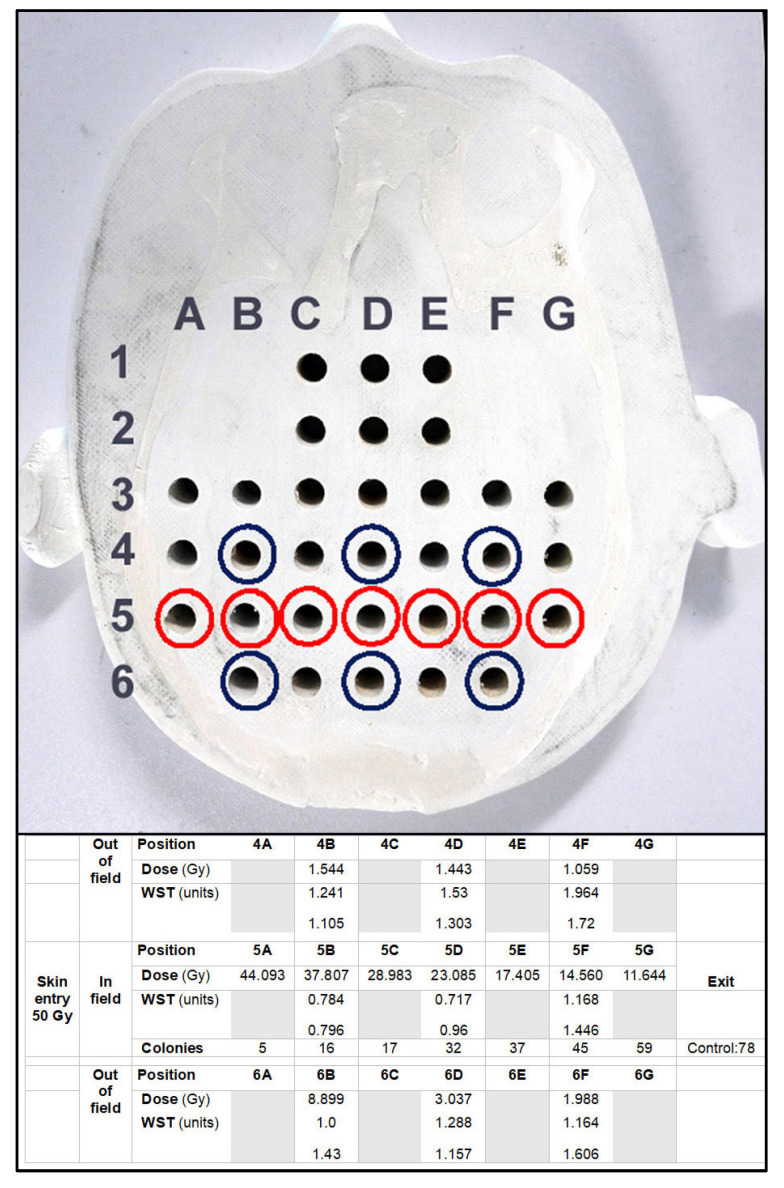
Measurement sites in the brain phantom and biological data obtained from the various positions within and outside of the irradiation field. The red circles mark the irradiation axis. Dosimetry probes and biological samples placed in this axis are located in the middle of a 20 mm wide irradiation field. The X-ray beam traversed the brain phantom in 5A to 5G direction (red circles). Since the distance between the individual measurement positions is approx. 15 mm, the axes 4A–G and 6A–G (blue circles) are outside of the treatment field and receive scattered irradiation only. For the positions in the axis number 6, the distance from the surface is slightly shorter than for the corresponding positions in axis number 4. There is less dose attenuation between skin entrance and target, which might explain the difference in measurement values between the corresponding out-of-field dose values of these two axes.

**Figure 5:**
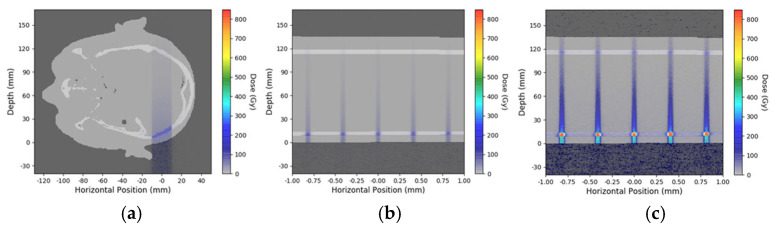
Geant4 simulation of depth-dependent dose decline in the brain slice phantom for broad-beam irradiation with a skin entrance dose of 50 Gy (**a**), an MRT peak dose of 50 Gy (**b**), and an MRT peak dose of 400 Gy at skin entrance (**c**).

Two copies of the brain slice phantom at pituitary gland level were produced, modified from the Alderson phantom including the pituitary gland. Dose measurements were conducted at skin entrance level and simulated for seven positions along the irradiation axis, including positions 5A to 5G (Figure 4). In addition, dose was simulated in six out-of-field positions (Figure 5). In a biomimetic context, this was performed to reflect radiobiological processes in organic tissue, where dose is produced and deposited outside the intended irradiation target by scattering processes. Since the phantom is produced from tissue-equivalent materials, we should look at very similar biological results as those seen in a real human brain. Between identical positions in the two new brain phantom slices, the values obtained varied by no more than 5%.

### 3.1. Depth-Dependent Dose Decrease After High-Dose-Rate Broad-Beam Irradiation, Correlated to Clonogenic Potential

The dose decreased by approx. 3.8 Gy/cm. This was reflected in the biological response, both in the metabolic activity as an equivalent of cell proliferation (WST) and in the numbers of lung cancer cell colonies counted. Clonogenic potential and metabolic activity in human lung cancer cells increased with increasing depth from surface, in response to the decrease in X-ray dose. Colony counts from the out-of-field positions were not significantly different from non-irradiated controls, except for the colonies raised from the sample in position 6B, where a higher incident dose had already been simulated, causing a higher degree of cell damage and, subsequently, a lower metabolic activity than in the corresponding position 4B.

### 3.2. Depth-Dependency of Metabolic Activity of Cancer Cells After MRT

**Figure 6:**
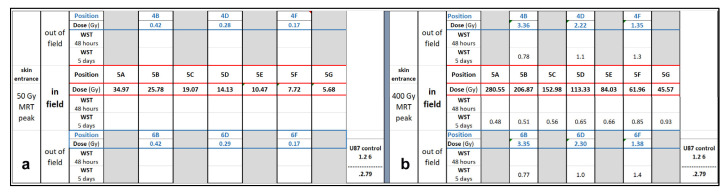
Simulation results for in-field and out-of-field dose for an MRT peak dose of 50 Gy (**a**) and 400 Gy (**b**) at skin entrance, correlated to WST results obtained in human brain cancer cells.

Not unexpectedly, the dose decline with increasing depth from surface correlated to an increase in metabolic activity as determined by WST. Similarly to the results seen in the preliminary WST experiment conducted in the 384-well plates, at 48 h after irradiation, there was no difference in the metabolic activity between irradiated and non-irradiated samples. At 5 days after irradiation, differences begin to appear (Figure 6). A likely explanation is that many components of the death cascade are not yet fully activated at 48 h after irradiation. The metabolic activity is lower in the position closest to the skin surface, where the highest X-ray doses were deposited.

**Figure 7:**
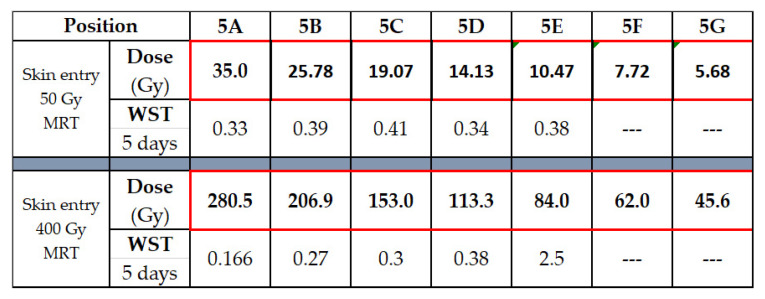
Simulated peak dose and WST values in lung cancer cells, 5 days after MRT.

The recovery of cancer cells from the agar column after irradiation is extremely labor-intensive. Furthermore, the accuracy of the cell count is biased by the experimenter’s dexterity and ability to extract the complete agar column from the tubes. In a long-term study, where the cancer cells can profit from the 3D environment by forming 3D cancer cell structures, this effort is justified. In a short-term experiment, where the cells are harvested shortly before the irradiation to be distributed in different dilutions after irradiation to serve a number of different analytic methods, this effort does not appear to be justified. We, therefore, abandoned this method in favor of irradiating cell pellets (Figure 7).

The MRT peak doses at skin entrance were 50 Gy and 400 Gy, respectively. The trend towards lower metabolic activity in positions close to the skin, as opposed to deeper in the ‘tissue’, is visible after MRT peak doses of 50 Gy and 400 Gy. Not unexpectedly, it is more obvious after irradiation with 400 Gy MRT peak dose. Interestingly, the WST values do not scale in the same way as the X-ray doses. In position 5A, for instance, the measured X-ray dose for 400 Gy at the skin entrance is approx. eight times as high as for 50 Gy, while the resulting WST value differs only by a factor of two. WST in non-irradiated controls was 0.466 (arbitrary units).

## 4. Discussion

In preparation of future human clinical trials with a focus on spatially fractionated radiotherapy, we have designed a biomimetic tool for translational research, incorporating biological parameters such as tissue shape and composition, which strongly influence the technical dose distribution and radiobiological response to irradiation. The result is an anthropomorphic brain slice phantom, which combines the advantage of commercially available phantoms, produced from tissue-equivalent materials, with the opportunity for the high sample throughput typical in radiobiological studies. Tissue slices including the regions of interest can be easily reproduced at a reasonable cost in as many identical copies as needed for a smooth-running experiment. Experimental time at major research facilities, such as a synchrotron, is awarded in a highly competitive process and, once obtained, it should be used wisely. Using synchrotron irradiation at high dose rates results in short irradiation times per sample. The whole irradiation procedure typically takes less than one minute, if the width of the irradiation target is not larger than the width of the available irradiation field. Populating the brain phantom with biological samples, on the other hand, usually takes longer than that. In such a scenario, the availability of several identical copies of the phantom, which can be modified to fit a specific experiment, helps to optimize the use of experimental time. The opportunity to fit biological samples into several phantoms in advance of an irradiation session assures a more rapid turnover of samples. While recently irradiated samples are removed from a phantom and its positions are re-filled with fresh samples, the next sets of samples are already irradiated in an identical phantom. We have tested this approach in several radiobiological studies at the synchrotron, where we have almost halved the turnover time between irradiated samples. These studies included a broad-beam irradiation technique at a high dose rate (photon FLASH) and spatially fractionated MRT at two different peak doses. Considering that synchrotron X-rays operate in the orthovolt range [21], the steep dose decrease within an organ along the irradiation axis and the correlated increase in the number of surviving cancer cell clones is hardly surprising. Interestingly, we also learned that the measured dose and biological response do not scale in the same way: while we see an inverse relationship between X-ray dose and cancer cell survival in all three experimental stratagems (50 Gy broad-beam FLASH, 50 Gy and 400 Gy MRT peak dose, respectively), it might take an 8-times increase in X-ray dose to achieve an approx. 50% reduction in cancer cell metabolic activity and proliferation, as represented by WST.

It was also interesting to see the extent to which even small differences in external morphology result in significant differences in dose deposition in the target (Figure 4). In clinical radiation therapy, the phantoms used are often geometrically shaped rather than anthropomorphically. Especially with spatially fractionated synchrotron irradiation, however, operating in the orthovolt range, already small anatomical variations may cause a difference in dose distribution which is significant enough to change the biological response of the tumor cells, as has been shown by the results of our study. Therefore, one potential clinical use for the biomimetic brain slice phantom would be the verification of a simulated treatment plan in future human MRT trials. In other words, the phantom can become a valuable component in the quality assessment procedure. Patient-specific brain slice phantoms for dose measurement, modeling the region of interest, could be produced based on CT scans or MRIs of individual patients, in a process similar to the one previously described in a human arm phantom [19].

The sharp depth-dependent dose decline and its subsequent decrease in tumor cell destruction shows that relatively high MRT peak doses will be required to achieve the desired therapeutic results in deep-seated targets (Figure 5). In the early times of radiation oncology, increasing skin surface doses to achieve therapeutically meaningful doses in deep-seated targets frequently resulted in high-degree radiodermatitis with skin ulceration. Even in those early times, spatial fractionation, with a grid deposited on the skin, dramatically increased the repair capability of the skin [22]. In MRT, we are equally taking advantage of spatial fractionation as a protective factor in normal tissue. It has been shown before that the fractionation at the micrometer range supports the preservation of normal tissue function, at high doses rates as well as in their absence [23,24,25].It should be pointed out that all the studies reported here were carried out using unidirectional irradiation techniques. As a nod to biomimetical concepts, future studies will follow clinical experience with stereotactic radiotherapy, where a high target dose is split into several smaller doses which enter the target from different directions. Thus, the normal tissue is traversed only by smaller X-ray doses, mostly below the tolerance threshold of normal tissue. In other words, normal tissue can repair the radiation-induced damage, whereas the dose deposited in the target region (the tumor) is high enough to induce permanent damage. With lower skin entrance doses, we expect that the effects caused by skull shape and bone thickness, as opposed to a simple spherical phantom, would be even more significant. The challenge in these stereotactic microbeam irradiation studies will be to precisely determine the isocentre of the irradiation field. Contrary to clinical stereotactic radiotherapy, where the gantry is rotated around the patient, at the synchrotron, the treatment beam position is fixed. This means that the patient needs to be rotated within the beam. While this might look fairly easy for a broad-beam technique like FLASH, achieving the required precision in treatment fields where the individual microbeams are only 50 μm wide will definitely be challenging. A biomimetic phantom will be a very welcome tool in these types of future studies, to improve the quality assurance process in experimental radiotherapy and future clinical studies. It can be used as a dosimetry tool to validate the simulated dose and reduce uncertainty associated with drift and daily fluctuations of the treatment beam [26]. Radiobiology studies can be conducted either in generic phantoms or in patient-specific phantoms (based on each individual patient’s CT scan), to identify the optimal treatment plan, where versions of the treatment plan offer significant differences in dose distribution. Uploaded to a commercial treatment planning system, the radiobiological response of the samples can be correlated to the dose distribution. Where time permits and tumor material is available, the radiobiological response could even be tested on samples generated from the patient’s own tumor. For such an approach, tumor tissue could be used as patient-derived organoids (PDOs) or, after homogenization of the tumor tissue, as 3D-bioprinted tumor structure [27]. PDOs can retain the heterogeneity of the original tumor including small blood vessels [28,29]. While this heterogeneity increases the likelihood of similar outcomes in vitro and in the patient, sample standardization and reproducibility might be easier to achieve in 3D-bioprinted structures. In either case, the integration of patient samples with a tissue-equivalent phantom, custom-printed from this patient’s CT scan, would combine several biomimetic aspects to decrease treatment uncertainty.

## 5. Conclusions

We have produced and validated a new biomimetic tool to improve the quality of translational research by taking into consideration key parameters which determine the response of cancer cells to irradiation, like the thickness and density of bone and soft tissue structures surrounding the irradiation target. Multiple identical copies can be produced at a comparably low cost. The easy technical approach and the low-cost production process make this type of anthropomorphic phantom a good tool for treatment plan validation as well as for radiobiology studies, especially for MRT or other high-dose-rate irradiation techniques with high sample throughput in a short time.

## Data Availability

Data are available upon request from the corresponding author (E.S.).

## Abbreviations

The following abbreviations are used in this manuscript:

AS	Australian Synchrotron
ESRF	European Synchrotron Radiation Facility in Grenoble, France
FDM	Fused Deposition Modeling
IMBL	Imaging and Biomedical Beamline
kV	kilo Volt
LINAC	Linear Accelerator
MRT	Microbeam Radiation Therapy
MV	Megavolt
PLA	Polylactic Acid
